# RNA interactome capture in yeast

**DOI:** 10.1016/j.ymeth.2016.12.008

**Published:** 2017-04-15

**Authors:** Benedikt M. Beckmann

**Affiliations:** European Molecular Biology Laboratory (EMBL), Meyerhofstrasse 1, 69117 Heidelberg, Germany; IRI for Life Sciences & Institut für Biologie, Humboldt-Universität zu Berlin, Philippstrasse 13, 10115 Berlin, Germany

**Keywords:** RBP, mRNA, Yeast, UV crosslinking, RNA labeling

## Abstract

RNA-binding proteins (RBPs) are key players in post-transcriptional regulation of gene expression in eukaryotic cells. To be able to unbiasedly identify RBPs in *Saccharomyces cerevisiae*, we developed a yeast RNA interactome capture protocol which employs RNA labeling, covalent UV crosslinking of RNA and proteins at 365 nm wavelength (photoactivatable-ribonucleoside-enhanced crosslinking, PAR-CL) and finally purification of the protein-bound mRNA. The method can be easily implemented in common workflows and takes about 3 days to complete. Next to a comprehensive explanation of the method, we focus on our findings about the choice of crosslinking in yeast and discuss the rationale of individual steps in the protocol.

## 1 Introduction

RNA-binding proteins (RBPs) display key functions in the cell such as RNA regulation, transport, translation or degradation by formation of dynamic ribonucleoparticles (RNPs) to regulate RNA fate and are thus fundamental for post-transcriptional gene regulation (PTGR, [23]). The need to identify RNAs bound to individual proteins has led to the development of high throughput techniques such as CLIP or CRAC (crosslinking and immunoprecipitation) from which diverse variations exists today [1], [7], [24], [29], [56], [57], [63]. In all of these methods, cellular RNA-protein interactions are crosslinked *in vivo* using UV irradiation to allow stringent purification of the RNPs prior to RNA sequencing. Historically, UV light of 254 nm wavelength has been used to crosslink RNA-protein interactions [8], [27], from here on called conventional crosslinking (cCL). An alternative protocol, photoactivatable-ribonucleoside-enhanced crosslinking (PAR-CL) requires incorporation of nucleotide analogs such as 4-thiouridine or 6-thioguanosine prior to UV exposure and crosslinking at 365 nm wavelength [21], [25].

Recently, the RNA interactome capture method has been developed to identify RNA-binding proteins associated to eukaryotic polyadanylated (poly(A)) RNAs [11], [2], [13]. After UV crosslinking, cellular poly(A) RNA is purified using oligo d(T) magnetic beads. The crosslinked RBPs are then subsequently subjected to identification by proteomic techniques such as mass spectrometry. To date, this approach has led to determination of the near-complete repertoire of RBPs in human HeLa cells [11], HEK293 cells [2], hepatocytic HuH-7 cells [5], HL-1 cardiomyocyte cells [36], mouse embryonic stem cells [35], macrophage RAW 264.7 cells [37], fly embryos [59], [53], in the worm *Caenorhabditis elegans* [40], in the parasite Plasmodium [9] and in *Arabidopsis thaliana* [38], [46].

The unicellular yeast *Saccharomyces cerevisiae* is a prominent model organism for studying genetics and eukaryotic cell biology such as the cell cycle due to its smaller proteome and transcriptome and well-established tools for gene editing. RBPs have been identified in yeast before, utilising *in vitro* techniques such as protein arrays combined with fluorescently labeled RNA probes [48], [54]. In a first attempt to map the RNA-bound proteome by *in vivo* crosslinking and poly (A) selection Mitchell et al. [44] identified 120 RNA-binders.

We used RNA interactome capture in yeast in combination with Orbitrap mass spectrometry to determine the mRNA-bound proteome of *S. cerevisiae* before [5]. We identified 678 high confidence RBPs; among them many proteins unknown to bind RNA, including enzymes involved in intermediary metabolism. Our approach has the advantage to ‘capture’ RNA-protein interactions *in vivo* using UV crosslinking. The resulting covalently crosslinked RNA-protein hybrids can then be purified under stringent wash conditions that remove secondary binders such as protein-protein interactions in RNPs. Here, we present a comprehensive description of the method itself, provide background for the individual steps and discuss the potential and limitations of the protocol.

## 2 Materials and methods

### 2.1 mRNA interactome capture in yeast

We included a step-by-step protocol in the Supplemental material for fast reference at the bench.

All buffers should be prepared using double distilled or ultra pure water, followed by autoclaving or sterile filtration.

#### 2.1.1 Culture conditions

We used *S. cerevisae* strain BY4741 for all experiments. Cells were grown in standard YPDA (yeast peptone dextrose adenine) medium or using a synthetic complete (SC) medium (yeast nitrogen base (YNB, BD Difco 291940), synthetic defined medium lacking uracil (SD-URA, Sigma Y1501), 120 μM Uracil, 1% glucose). Liquid yeast cultures were inocluated with an OD_600_ of 0.01–0.05 and grown at 30 °C while shaking at 160 rpm. For RNA interactome capture experiments, 5 ml YPDA preculture was inoculated from a single colony on an agar plate and grown over night. The next day, a 1 L culture of SC-medium_120μM Ura_ was started with the preculture. Because we generally include a non-irradiated sample as negative control, for each experiment we have at least two yeast cultures.

#### *2.1.2* *In vivo* RNA labeling

Yeast in SC_120μMUra_ medium were grown to an OD_600_ of 0.5 and 4-thiouracil (4tU, Sigma 440736) was added to a final concentration of 500 μM using a 50 mM 4tU stock solution (dissolved in dimethyl sulfoxide, DMSO). We used 4-thiouracil (4tU) and not 4-thiouridine (4SU) which is routinely used for *in vivo* RNA labeling in mammalian cell culture experiments (see discussion). Note also that YPDA medium can be used as previously shown [50] for RNA labeling in yeast using 4tU. Cells were grown for another 3 h to permit several cell divisions and incorporation of 4tU into RNA with a low turnover. Cells were then harvested by centrifugation (4000 rpm; 15 min; 4 °C) and the cell pellet was immediately dissolved in 40 ml cold double-distilled H_2_O (ddH_2_O). From this moment on, cells were kept on ice whenever possible.

#### 2.1.3 UV crosslinking

Next, we spread the cells from one sample evenly onto two standard Petri dishes (10 cm diameter). The dishes were placed on a tray with ice and Ultraviolet crosslinking was performed in a Spectrolinker device (Spectronics, XL1500F/A) emitting UV light at 365 nm wavelength (PAR-CL). The cells were placed in ∼ 5 cm distance to the UV bulbs. We used energies from 0.72 to 7.2 J/cm^2^ which roughly corresponded to 3 and 30 min or irradiation time, respectively. Every 10 min, irradiation was interrupted and the dishes were carefully rocked for 30 s to allow cooling of the cells and a redistribution of cells in the liquid layer. Finally, cells were collected from both dishes using a 20 ml glass pipet by carefully washing off the cells with the liquid in the dish in a 50 ml tube on ice. The non-crosslink control was kept on ice during the irradiation of the other samples.

#### 2.1.4 Cell lysis

Cells were pelleted by centrifugation (4000 rpm, 5 min, 4 °C) and the pellet was dissolved in 2 ml lysis buffer (20 mM Tris pH 7.5, 500 mM LiCl; 0.5% LiDS; 1 mM EDTA; 5 mM dithiothreitol (DTT); 1 protease inhibitor mix (EDTA-free, Roche, 11873580001); 1 mg/ml RNasin; 200 mM vanadyl-ribonucleoside complex, VRC). The mixture was distributed over several 2 ml screw-cap tubes containing an equivalent of 300 μl acid washed glass beads and cell lysis was performed using a Fast Prep device (MP Bio; 6 m s-1; 5 × 50 s bursts with 20 s pausing in between) in a cold room. The lysate was cleared of cell debris by centrifugation (12,000 rpm; 2 min; 4 °C) and the supernatants were transferred to a 50 ml tube on ice. At this point, the lysate can be snap-frozen and stored at −80 °C for up to one week before continuing the protocol.

#### 2.1.5 RNA interactome capture

Oligo d(T) beads (NEB S1419) were pre-equilibrated with lysis buffer: we washed 1 ml of beads 3 times with 1 ml of lysis buffer using a magnet to remove the supernatant. The yeast lysate in the 50 ml tube was filled up to 25 ml with lysis buffer and an aliquot (100–500 μl) was saved at -20 °C as input sample. We added an equivalent of 1 ml oligo d(T) beads (pre-equilibrated) per litre of starting culture. Binding of the beads to RNA was then performed in an overhead rotating device in the cold for 1 h using a rotation of 20 rpm. Separation of the magnetic beads was also at 4 °C for until the supernatant was clear (which took usually around 30 min) and the supernatant was stored on ice for a second round of binding. All subsequent steps were carried out in a 15 ml tube at room temperature but buffers were kept on ice. The beads were then washed in 10 ml lysis buffer by rotating overhead for 5 min and magnetic separation until the supernatant was clear. We then repeated this wash step again two times in wash buffer 1 (20 mM Tris pH 7.5, 500 mM LiCl; 0.1% LiDS; 1 mM EDTA; 5 mM DTT), two times in wash buffer 2 (20 mM Tris pH 7.5, 500 mM LiCl; 1 mM EDTA) and two times in low salt buffer (20 mM Tris pH 7.5, 200 mM LiCl; 1 mM EDTA) while discarding supernatants inbetween. An important hallmark of the experiment can usually be observed when performing washes with wash buffer 2: the UV-irradiated sample only should form a transparent “halo” in the colour of the beads surrounding the opaque oligo d(T) beads after magnetic separation indicating successful crosslinking of proteins to the selected poly (A) RNA. Finally, beads were resuspended in 500 μl elution buffer (20 mM Tris pH 7.5; 1 mM EDTA), transferred to a 1.5 ml tube and incubated at 55 °C for 10 min. After magnetic separation, the supernatant (eluate) was collected and stored at -20 °C. The used beads were recovered in lysis buffer and washed 3 times to re-activate and pre-equilibrate the beads for a second round of interactome capture using the supernatant from the first oligo d(T) binding. Due to loss of magnetic beads during the first round, we routinely added another 200 μl freshly pre-equilibrated beads and repeated the selection procedure exactly as in the first round. The second eluate was then added to the first (the majority of poly (A) RNA and proteins are recovered already in the first round). We did not observe a significant increase in RBP yield when performing a third round of interactome capture in yeast.

### 2.2 Downstream applications

At this point, eluates from the oligo d(T) selection consist of crosslinked proteins (RBPs), crosslinked poly (A) RNA and non-crosslinked poly (A) RNA from yeast.

#### 2.2.1 RNase digestion

To be able to analyse proteins, removal of the covalently bound RNA is highly advisable (e.g. for western blotting or proteomics applications). We perform RNA digestion by adding 250 μl of 5× RNase buffer (100 mM Tris-HCl pH 7.5, 75 mM NaCl), 200 U RNase A (Sigma-Aldrich, R4642) and 200 U RNase T1 (Sigma-Aldrich, R1003). Digestion was done for 1 h at 37 °C before placing the sample on ice. We then concentrated the RBPs using an Amicon Ultra centrifugal filter (0.5 ml; 3 kDa cut-off; Millipore UFC901024) according to the manufacturers instructions, resulting in 25–30 μl of RBPs. These can be directly subjected to SDS-PAGE, mass spectrometry sample preparation, etc. Note however that the two RNases are retained in the sample in relatively high amounts; however because of their small size and the subsequent limited number of peptides derived from the these after trypsin treatment, RNases do not interfere with the downstream mass spectrometric analysis, at least under our experimental conditions. SDS PAGE and silver staining were performed using standard techniques as described before [5].

#### 2.2.2 Proteinase K digestion

When investigating the RNA after oligo d(T) capture, Proteinase K digestion can be used to remove the crosslinked polypeptides. We performed enzymatic proteolysis by adding 250 μl of 5 x PK buffer (50 mM Tris-HCl pH 8.0; 5 mM EDTA; 2.5% SDS) and 0.4 mg/ml proteinase K (Roche) at 50 °C for 1 h. We then performed RNA extraction using either the single step method [15] or its commercially available version (Trizol, according to the manufacturers instruction; Thermo Fisher 15596026).

Finally, I would like to highlight that both strategies, removal of proteins or RNA, will result in samples that still contain covalently crosslinked residues from the partner molecule; neither RNase nor protease digestion completely removes the crosslinked RNA or peptide(s), respectively. This should be taken into consideration when planning downstream analyses such as (q)RT-PCR which is sensitive to such crosslinking sites [25].

#### 2.2.3 Radioactive scintillation counting

Human beta globin mRNA carrying a poly (A) tail of 62 residues was *in vitro* transcribed using T7 RNA polymerase (Thermo Fisher 18033-019) using the protocol provided by the manufacturer, 5′-end labeled using T4 polynucleotide kinase (PNK, NEB M0201S) and gamma ^32^P ATP (Hartmann Analytics SRP-201). After purification, the RNA was added as spike-into whole cell lysates generated with lysis buffer +/− LiDS. After magnetic separation, flowthrough of each intermediary step of the interactome capture protocol was collected and beta radiation was measured using a Perkin Elmer Liquid Scintillation Analyzer (TriCarb 2800 TR) and the QuantaSmart Software.

For protein labeling, 100 ml yeast cultures were grown to an OD_600_ of 0.5 and ^35^S Methionine (Hartmann Analytics MS-103) was added to the culture. After 1 h, cells were lysed as described in buffer containing LiDS and 200 μl lysate was mixed with 100 μl oligo (T) beads. Flowthroughs after magnetic separation were mixed with 2.5 ml scintillation liquid (Perkin Elmer Ultimate Gold High Flash point KSC cocktail 6013327) and radioactive signals were measured.

#### 2.2.4 Western blotting

SDS-PAGE and Western blotting were performed according to standard protocols. Antibodies used: PUB1 and PAB1 (1:3000, kind gifts from Maurice Swanson), tubulin (1:3000; Abcam ab6161), histone H4 (1:1000; Cell Signaling 2592).

#### 2.2.5 Real-Time PCR

Total RNA from cells was isolated using the single step method [15] and oligo d(T)-isolated RNA was proteinase K digested as described above to remove crosslinked polypeptides. We then generated cDNA using random hexamers and SuperScript II reverse transcriptase (Thermo Fisher 18064014) according to the manufacturers instructions. Quantitative PCR (qPCR) was then performed using specific primers against 18S rRNA (forward: TTGAATGAACCATCGCCAGC, reverse: TATCTGGTTGATCCTGCCAG), ACT1 (forward: CGTCTGGATTGGTGGTTCTA, reverse: GATGGACCACTTTCGTCGTA) and TDH3 (forward: CGGTAGATACGCTGGTGAAGTTTC, reverse: TGGAAGATGGAGCAGTGATAACAAC) in a 7500 Real time PCR system (Applied Biosystems) and normalised against a Renilla luciferase spike-in control mRNA, added during lysis (forward: GAATTTGCAGCATATCTTGAACCAT, reverse: GGATTTCACGAGGCCATGATAA). The external Renilla spike-in was used to normalise the input levels of the three genes inbetween biological replicates. For quantification, we used the delta delta Ct method.

#### 2.2.6 Mass spectrometry

Proteomic detection of the purified RBPs can be achieved by mass spectrometry. To determine the yeast RNA interactome, we first digested purified proteins (crosslinked samples as well as the non-crosslinked control) with Lys-C and trypsin. Then, sample and control peptides were differentially labeled using stable dimethyl isotopes. After mixing sample and control, peptides were split into 12 fractions according to their pH (ranging from pH 3–10) using a 3100 OFFGEL fractionator (Agilent) and the 12 fractions were finally analysed by LC-ESI-MS/MS using an Orbitrap Velos Pro (Thermo Fisher). Identification of peptides/proteins was performed using MaxQuant [16]. For further details, see [5].

### 2.3 Statistical analysis of high throughput RBP detection

If the interactome capture approach is not used to investigate individual RBPs (e.g. by western blotting) but to determine the RNA-bound proteome of yeast, the usage of mass spectrometry (see above) for unbiased detection of RBPs is necessary. However, after proteomics, a thorough statistical analysis is inevitable. I will discuss our approach to determine the RNA interactome capture of *S. cerevisae* [5] here (Fig. 3A) with the bioinformatic programs we used to conduct this analysis. Please note that this pipeline could be followed using a plethora of different tools.

#### 2.3.1 Protein enrichment after crosslinking to RNA vs. non-crosslinked samples

• For all proteins which were successfully identified by mass spectrometry, we collected the obtained fold-changes of proteins identified in crosslinked over non-crosslinked samples from all three repetitions. Our dimethyl labeling approach allowed us to directly obtain those ratios; if label-free proteomics techniques are used, ratios have to be generated from the quantified protein levels first (see [11]). Standard spreadsheet calculation programs such as Excel are useful to store the data on this level. It is good practice to store the ratios as log enrichment: 0 = no difference between crosslinked and non-crosslinked; < 0 = enriched in the non-crosslinked sample; > 0 = enriched in the crosslinked sample
• Next, the means of the ratios from the individual biological repetitions were generated.

#### 2.3.2 Statistical analysis

For the next steps, we used R/Bioconductor and the Limma package [47]. For a hands-on introduction to statistical analysis on biological data in R, I recommend [30]. Given that we are analysing hundreds of proteins with different abundance levels, RNA-binding modes and crosslinking probabilities, we are dealing with a multiple testing problem.

• Inferring significance: We first tested differential enrichment using an empirical Bayes moderated *t*-test. This moderated *t*-test is advantageous over the normal *t* statistic as it is less influenced by under-estimated sample variances [51].
• P-values alone are of limited use when interpreting high-dimensional data since many features could influence the identification and levels of recovered RBPs. Multiple testing was thus performed to estimate the false discovery rate (FDR) using the method of Benjamini and Hochberg. The resulting adjusted value was labeled ‘p adj’: all proteins within FDR of 0.01 were selected as RNA interacting proteins. We thus assume that 1% of the 678 RBPs are false positive hits.

#### 2.3.3 Visualisation of the statistical analysis

We mainly employ two types of plots to visualise outcome of the statistical analysis:

• We used scatter plots to compare enrichment of crosslinked proteins from individual repetitions and assess reproducibility inbetween biological replicates. We coloured dots of proteins which, after multiple testing of all replicates as described before, were within the 1% FDR range (p adj < 0.01).
• A very useful approach to visualise all replicates in summary is to display the data in a volcano plot. Instead of the individual log-fold ratios, the mean log-fold ratio is plotted on the X-axis. On the Y-axis is the negative of the log of the adjusted p-value (−log2 p adj). This representation allows to quickly inspect how many proteins are enriched in the non-crosslinked control (<0; left side of the plot) and which are enriched as RBPs after crosslinking (>0; right side of the plot). We again coloured all proteins with an FDR of 0.01 or smaller.

### 2.4 RNA interactome capture troubleshooting

- We note that the ratio of lysed cells compared to the volume of oligo d(T) magnetic beads is crucial for a successful experiment. We routinely used 1 ml of beads per 1 L of yeast culture.
- Since our method relies on RNA labeling, it is important to test efficiency of RNA labeling during setup of the protocol. We tested *in vivo* RNA labeling with 4tU using a dot blot approach [19] to ensure incorporation of the modified uridine into nascent RNA.
- In some trials, white precipitates formed during the initial binding of poly (A) RNA with beads in the cold. These are most likely larger complexes formed by chromosomal DNA and proteins. Increasing the volume during the binding step with lysis buffer is then advisable to dissolve those precipitates.
- During washes, we repeatedly noticed a brownish layer forming at the surface of the tubes. This is a result of beads sticking to the inner side of the tubes and can lead to significant loss of beads and purified RNA-protein complexes. Addition of 0.05% NP-40 to wash buffer 1 and wash buffer 2 helps to reduce the loss of beads. However, NP-40 may severely impair downstream applications such as some mass spectrometry protocols and we thus restrained from adding it to the low salt wash buffer or elution buffer.

### 2.5 Notes on timing of the protocol

Yeast RNA interactome capture is a relatively straightforward protocol that does not require special equipment or devices that cannot be found in a standard molecular biology laboratory, maybe with the exception of the 365 nm UV bulbs and a magnet to separate beads in 50 ml volume. It takes 3 days to perform the complete protocol:

- Day 1: Cell growth, RNA labeling & UV crosslinking
- Day 2: 2 rounds of oligo d (T) binding and washing
- Day 3: RNase digestion of the eluates, SDS-PAGE & visualisation of the RBPs

However, the method requires a lot of hands-on time, esp. during day 2 in which 5 min wash steps alternate with magnetic separation (30–5 min).

### 2.6 Methods to validate candidate RBPs

After high throughput screens for potential novel RBPs, our described analysis (Section 2.3) results in a list of candidate RBPs. Such newly-detected RNA-binding proteins, or at least a subset, should be validated to be of *bona fide* RNA-binding capacity. Several methods exists from which I would like to highlight three:

- Western blotting: This is maybe the most straightforward approach to validate a protein to bind RNA. Immunoblotting can be performed after oligo d(T) selection and RNA digestion as described above. However, since this approach is basically the interactome capture protocol again and is likewise based on UV crosslinking and oligo d(T) selection, this validation technique is best used to validate the mass spectrometry part of a high throughput screen for RBPs. Also, availability of antibodies might limit the usefulness of western blotting as validation technique. In summary, it is not advisable to solely rely on western blotting to validate novel candidate RBPs
- PNK assay: Following *in vivo* UV crosslinking, individual candidate RBPs are immunoprecipitated with specific antibodies. After limited RNase digestion, covalently crosslinked shortened RNAs are 5′ phosphorylated with ^32^P-gamma ATP (radioactive isotope) using polynucleotide kinase (PNK). Next, the complex is analysed by SDS-PAGE. A radioactive signal detected at the molecular mass of the protein then indirectly proofs RNA-binding capacity. This assay has the advantage that the purification of the complex does not rely on oligo d(T) purification but on immunoprecipitation of the candidate RBP. Also, since all types RNA can be phosphorylated by PNK in principle, this validation approach does not distinguish which RNA targets are bound by the RBP.
- CLIP-Seq: This could be seen as an extension of the PNK assay. Instead of radioactively labeled RNA as read-out, the crosslinked RNAs are identified by next generation sequencing. CLIP-Seq will yield much information on the RNA species of the RNP complex. However, this type of analysis also requires biological replicates and downstream bioinformatics analysis which could render it impossible to validate a larger number of candidate RBPs due to cost and time investment.

In the study in which we determined the yeast interactome [5], we used a combination of all of the three validation techniques.

## 3 Results

### 3.1 Purification of yeast RNA-binding proteins

**Fig. 1:**
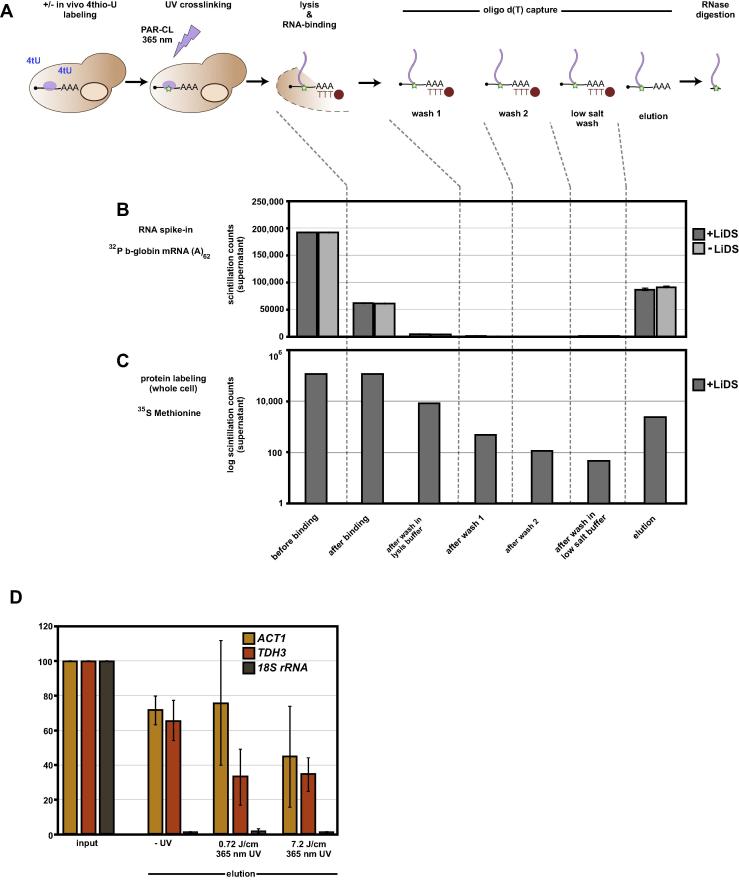
Setup of mRNA interactome capture. A) Detailed schematic of the interactome capture protocol. B) mRNA recovery of a poly-adenylated 32P-body-labeled beta-globin RNA. Scintillation counts in lysates or supernatants of each step of the protocol are shown. After addition of the spike into whole cell lysates, capture was performed +/− LiDS in lysis and wash buffer. C) Protein recovery of 35S-Methionine-labeled proteins. Scintillation counts of 35S levels of supernatants are shown for individual steps of the protocol. D) qRT-PCR of actin (*ACT1*), GAPDH (*TDH3*) and ribosomal RNA (18S rRNA) from cells lacking UV irradiation (-UV), 0.72 or 7.2 J/cm^2^ of 365 nm UV light exposure. Input was adjusted to 100% and recovered mRNA levels are shown as fraction of the input. Error bars in panels B, C and D represent standard deviation (SD), n = 3.

RNA interactome capture (Fig. 1A) consists of a series of steps in which growing yeast were first *in vivo* RNA labeled using 4tU and then UV-crosslinked by irradiation with light at 365 nm wavelength. Next, the cells are lysed with glass beads in a buffer containing 0.5 M salt, the anionic detergent lithium dodecylsulfate (LiDS) and a reducing agent (dithiothreitol, DTT). Such stringent conditions can be applied only as the initial UV irradiation resulted in a covalent crosslink inbetween RNA and interacting proteins. Then, poly (A) RNA was selected by binding to oligo d(T) magnetic beads and the captured (m)RNA (including the crosslinked, denatured peptides) was washed in three different buffers prior to elution of the RNA from the beads.

To be able to assess RNA recovery throughout the procedure, we tested each individual step in the protocol first for recovery of a ^32^P-labeled spike-in RNA to ensure that buffer conditions do not impair the poly (A):oligo d(T) interaction; an *in vitro*-transcribed, body-labeled, human beta-globin mRNA carrying a poly (A)_62_ tail was incubated with whole cell lysate and levels of radioactive signal were determined from supernatants/flowthroughs of each intermediate step by scintillation counting. Since each wash step consists of two consecutive washes in the same buffer, we show pooled signals from both repetitions (Fig. 1B). The presence of the detergent does not affect the efficacy of recovery of the labeled RNA probe as elutions from lysates in presence or absence of LiDS are comparable. Also, once bound, the labeled RNA is efficiently retained on the beads as we barely detect signals in the flowthroughs. These findings were further confirmed by qRT-PCR from actin (*ACT1*) and GAPDH (*TDH3*) mRNA as well as 18S rRNA as non-polyadenylated, negative control (Fig. 1D). Recovery of individual cellular mRNA species ranges from 35 to 70% of the input, comparable to previous studies [11], [35], whereas rRNA is efficiently depleted in the sample. Interestingly, we observe decreased recovery of transcripts after crosslinking compared to the non-crosslinked sample. This is also reflected by standard 260 nm UV concentration measurement of purified RNA using a Nanodrop spectrophotometer (data not shown); RNA concentration from non-crosslinked samples is usually ∼1.6-fold higher compared to the crosslinked samples. We attribute this difference to a greater loss of magnetic beads when RNA is crosslinked to proteins (see troubleshooting Section 2.3). Next, we tested for wash efficiency and protein recovery by labeling proteins cell-wide using ^35^S-Methionine before performing interactome capture (Fig. 1C). Each step of the protocol is accompanied with a significant drop of radioactively labeled proteins resulting in scintillation counts close to background levels in the final wash step. As expected, only a small fraction of the labeled cellular proteins could be recovered after elution as UV crosslinking has been previously shown to display low overall crosslinking efficiency (see discussion): about 1% of the labeled proteins are captured by our method (Fig. 1C).

**Fig. 2:**
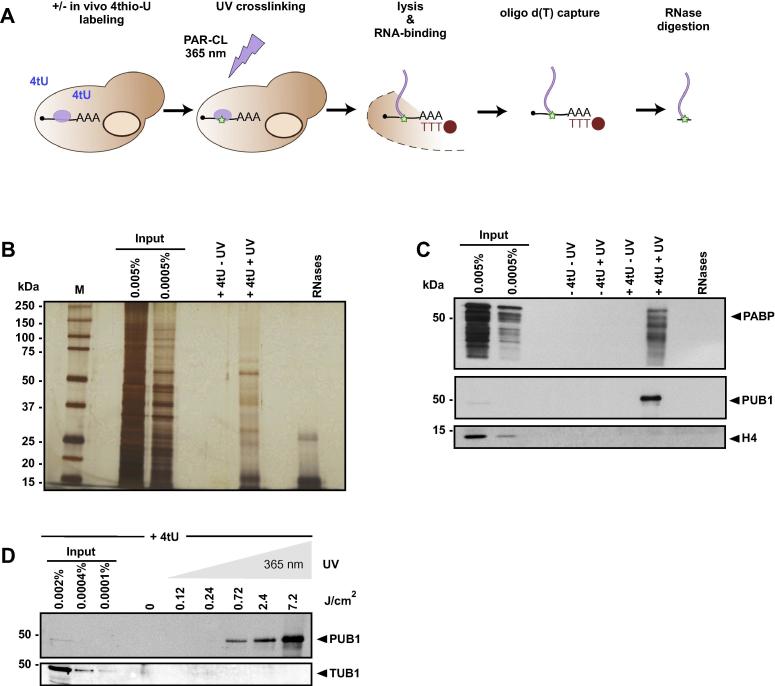
Capture of mRNA-binding proteins. A) Schematic of mRNA interactome capture. B) Silver-stained SDS-PAGE of yeast cell lysate (Input) and eluates after interactome capture from 4tU-labeled cells. C) Western blot analysis of mRNA interactome capture; cell lysate (Input) and eluates from capture experiments (PAB1, polyA binding protein; PUB1, polyU binding protein 1; H4, histone 4). D) Western blotting of series of UV crosslinking ranging from 0 to 7.2 J/cm^2^.

For protein analysis, we digested RNA after elution from oligo d(T) beads using RNase A and RNase T1 as the covalently crosslinked nucleic acids add a huge mass to the denatured proteins, which would prevent size-dependent separation of the RBPs during gel electrophoresis. We routinely analysed samples +/− crosslinking using SDS-PAGE and silver staining (Fig. 2B). When comparing purified samples with the input controls, we observe a specific pattern of proteins (RBPs) captured by our method which do not resemble the global distribution of proteins in whole cell lysate. Also, we do not capture proteins if omitting the crosslinking step (+4tU -UV), demonstrating that the stringent wash conditions indeed remove unwanted, crosslink-independent protein contaminants. We further performed immunoblot analyses, adding additional controls from cells that were not *in vivo* labeled (Fig. 2C; -4tU lanes). However, only the combination of *in vivo* RNA labeling using 4tU together with UV crosslinking at 365 nm results in capture of well established RBPs such as the poly (A) binding protein (PAB1) or poly U binding protein (PUB1). These results demonstrate that proteins recovered by interactome capture are enriched because of the interaction with RNA and that the crosslinking technique is indeed specific for proteins immobilised on RNA.

During the setup of the protocol, we investigated the correlation of irradiation energies during UV crosslinking and RBP recovery. As expected, we detected higher yields of captured RNA-binders such as PUB1 when extending crosslinking times (Fig. 2D). However, even after 30 min of irradiation with 365 nm, we do not observe contamination with abundant non-RNA binding proteins such as histone H4 or tubulin which we used as negative controls.

## 4 Discussion

The RNA interactome capture approach has, in combination with proteomic analysis by mass spectrometry, been used for system-wide mapping of (m)RBPs in different cell lines and organisms. Hundreds of novel RBPs have been identified by this technique; many of them were unexpected to bind to RNA, contain no recognisable RNA-binding domain [12] and the functionality of binding to RNA remains enigmatic for many of those proteins for the time being [5]. In this respect, it is important to highlight that the RNA interactome capture approach relies on irradiation of biological samples with UV light which, as discussed before, results in a zero-distance crosslinking event and formation of a covalent bond between a nucleotide of the RNA and a peptide from an adjacent protein. All proteins which can be crosslinked by this approach are RNA-binding proteins in the true meaning of the word “binding”: those proteins physically contact RNA and will be captured by the presented protocol. At this point, we cannot infer any particular role or function of a so-captured individual protein in RNA biology. Hence, we have two deal with the following consequences: since each protein could in principle be close in space to a random RNA at a given time and be crosslinked along with other, well-established RBPs, it is important to carefully control interactome capture experiments using biological replicates and including negative control samples when performing quantitative downstream applications. For the same reason, we determined the enrichment of crosslinked proteins after oligo d(T) selection over the non-crosslinked ones [5]. Selecting statistically significant proteins originating from true biological replicates in combination with a quantitative assessment of enrichment by UV crosslinking (others used a non-poly (A) control for the same purpose [40]) is thus important to protect against wrong assignment of a protein as *bona fide* RBP. Still, many of the so-identified proteins lack a role (yet) in RNA biology. However, not all of these novel RBPs must necessarily contribute to regulation of (m)RNA. We previously discussed that certain RNAs have evolved to bind and/or regulate proteins [4] or act as substrate for protein activity as established for Toll-like receptors or other immune response proteins such as RIG-I [32], [62]. RNA acting as a scaffold for protein assembly as in the case of snRNPs or snoRNA [60], [58] or to direct proteins to a specific target as shown for siRNA and argonaute proteins are other examples of the many facettes in which RNA and proteins interact [41]. Taken together, we believe that the interactome capture approach represents an image of the cellular diversity of RNA-protein interactions beyond canonical RNA-binding and RNA-regulating proteins.

### 4.1 Comments on individual steps of the method

#### *4.1.1* *In vivo* labeling and UV crosslinking

In interactome capture approaches in mammalian cell culture, 4-thiouridine (4SU) was used for RNA labeling instead of 4-thiouracil (4tU) [11], [5]. Unlike other cells from various organisms, *S. cerevisiae* has no uptake mechanism for 4SU from the surrounding medium [42]. This shortcoming can be overcome by expression of a heterologously expressed human equilibrative nucleoside transporter (hENT) [28], [42]. Most conveniently, 4tU (dissolved in DMSO) is easily taken up and incorporated into RNA by yeast wild-type cells. To minimise any potential background caused by the heterologous expression of a transporter, we continued to use 4tU in wild type cells when labeling RNA in yeast *in vivo*. In CV-1 monkey kidney cells, high concentrations of 4SU and UV irradiation have been shown to interfere in protein biosynthesis [22] and inhibition of rRNA production was found in human U2OS cells after *in vivo* RNA labeling using >100 μM 4SU [10]. Under such conditions, we can hence expect to induce secondary, unwanted effects and/or RNA-binding events which are stress-related and do not represent the normal physiological context of the cell. When testing yeast for 4tU incorporation for up to 3 h, we used concentrations from 100 μM to 1 M (f.c.) 4tU; growth curve analyses did not yield significant differences between the 4tU-containing cultures, a DMSO-only control and medium only (data not shown). However, pulse labeling of the yeast transcriptome with 4-thioU has been achieved in about 6 min [42] and even 2.5 min [3].

After performing yeast interactome capture, we treated the significantly enriched recovered proteins as candidate RBPs, following the rationale that these proteins were crosslinked initially to RNA. Thus, it is of high importance to control for residual proteins and carry-over of abundant cellular proteins which are not related to RNA-binding. We already discussed the necessity to include a non-crosslinking control as this sample includes the background of proteins (potential false positives). In this respect, another advantage of UV crosslinking originates from the covalent bond which is formed upon irradiation [20], [8], [27]. The covalent bond is resistant to chaotropic and denaturing conditions, allowing to use high salt buffers, detergents such as LiCl and reducing agents (DTT) during the interactome capture protocol. Such stringent conditions are commonly used to remove proteins during purification of nucleic acids. We routinely performed SDS-PAGE and silver staining to check for protein contaminations in the non-crosslinked control. The fact that we fail to detect even highly abundant proteins in this sample using the sensitive silver staining technique or in western blots demonstrates the selectivity of the method for crosslinked proteins (see Fig. 2B, C). Another advantage of using UV light for crosslinking is the possibility to “freeze” RNA-protein interactions in relatively short time: permitting to generate a snapshot of the RNA-bound proteome of the desired state of the cell.

Other interactome capture studies performed in mammalian cell culture benefitted from cell lines which grow adherent on the surface of the cell culture dish [11], [2]. Those cells form a monolayer which is highly advantageous for UV crosslinking as this allows maximal penetration of each cell in the respective dish with the same amount of UV light during irradiation after removal of the growth medium. Suspension cultures such as yeast on the other hand suffer from a severely reduced crosslinking efficiency compared to adherent-growing cells as yeast has to be spread out in buffer or medium. We can assume a series of complications: first, we expect a reduced penetration of all cells with UV light due to absorbance of the 365 nm light by the liquid (water, PBS, buffer, etc.). Second, since cells are not grown in a monolayer during irradiation, stacking of cells upon each other might absorb UV light and “shield” cells underneath from irradiation which finally will result in an uneven distribution of crosslinking events comparing cells at the surface and cells at the bottom of the dish. To compensate for these issues, we use an extended irradiation energy (up to 30 min of *in vivo* crosslinking; corresponding to ∼7.2 J/cm^2^; others used energies up to 12 J/cm^2^ [17], [50]) and repeated gentle shaking of the dish to allow cooling as well as repositioning of the yeast cells.

#### 4.1.2 Selection of poly (A) RNA

Despite the fact that we sometimes refer to our method as “(m)RNA interactome capture”, we feel that it is important to remind the reader that we are actually determining a poly (A) RNA interactome since selection of mRNA is facilitated by usage of oligo d(T) beads during stringent wash steps. Although the majority of mature eukaryotic mRNA is poly-adenylated, some proteins such as histones are translated from poly (A) free mRNA [39]. Proteins interacting with such mRNA can thus not be captured. On the other hand, also other types of RNA may carry poly (A) tails: non-coding RNA (ncRNA) transcribed by RNA polymerase II such as cryptic unstable transcripts (CUTs) will likewise be poly-adenylated [61], [55]. Poly-adenylation of RNA was described as a degradation intermediate of RNA processed by the exosome [6], [49] and it was reported that a small fraction of nucleolar ribosomal RNA in yeast can carry a poly (A) tail [31], [34]. This is also reflected in our dataset as we find ribosomal proteins among the enriched RBPs [5]. Interestingly, we observed a relatively low yield of mitochondrial RBPs, particularly in comparison to mammalian interactomes. Mitochondrial RNA is generally not poly-adenylated during *de novo* transcription in eukaryotes; however animal cells harbour the mitochondrial poly (A) polymerase PAPD1 which was found to add a poly (A) tail of around 50 adenylates to RNA derived from mitochondrial genes [14]. As this particular additional polymerase is missing in the yeast *S. cerevisiae,* RBPs interacting with mitochondrial RNA species could not be selected in our approach in yeast, providing an explanation for the lower number of captured mitochondrial RBPs.

### 4.2 Determinants for successful mRNA interactome capture

In our efforts to understand the composition of yeast RBPs identified by mRNA interactome capture and the key factors that determine the outcome of efficient RBP recovery, we analysed newly found high confidence candidate RBPs along with the well established RNA-binders for various features. We found RBPs with various binding modes and with diverse specificities for RNA. RNA-binders with well-defined RNA target sites such as the poly (A) binding protein (PAB1, recognising poly (A) stretches), as well as helicases which can be considered to move along RNA and thus do not bind a particular motif or site stably, to name only a few [5]. Looking at domain structures, we find that identified RBPs are very diverse, comprising different RNA-binding domains (RBDs) such as the RNA recognition motif (RRM), zing finger domains, heterologous nuclear RNP K-homology domain (KH) as well as proteins that lack RBDs. In-depth analyses of the RNA interaction sites within RBPs indeed confirmed that RNA-binding is not limited to RBDs but RNA-binding sites were mapped to unstructured regions in RBPs as well, displaying the plurality of RNA-binding modes [33], [12].

#### 4.2.1 Crosslinking determines recovery

When assessing the recovery of well-established RBPs in yeast such as PUB1, KHD1, PAB1 (Fig. 2 C in [5]) or in human HuH-7 cells (e.g. PTBP1, CSDE1 [5]) by comparing the protein amounts of input and interactome capture-purified proteins in western blots, we could roughly estimate that recovery is often <1% and only in rare cases more than 5% of an individual RBP was captured (such as human MOV10 [52]). These low crosslinking efficiencies are consistently observed throughout different experimental setups in many organisms. When performing CLIP or CRAC-type experiments to determine the RNA targets of a single RBP, short irradiation accompanied with lower crosslinking yields can be compensated for by amplification of the bound RNA during (RT)-PCR which is routinely used in RNA-Seq protocols. For interactome capture however, no such downstream amplification is possible as mass spectrometry (or other protein analysis techniques) is applied on the purified proteins directly to identify RBPs. To be able to overcome this obstacle, we employed irradiation with 365 nm UV light for up to 30 min (7.2 J/cm^2^) [17] and prior to mass spectrometry, the sample was fractionated in 12 subsets (isoelectric focusing; fractions of the peptides ranging from pH 3–10 [5]), thereby increasing the “depth” of peptide identification.

**Fig. 3:**
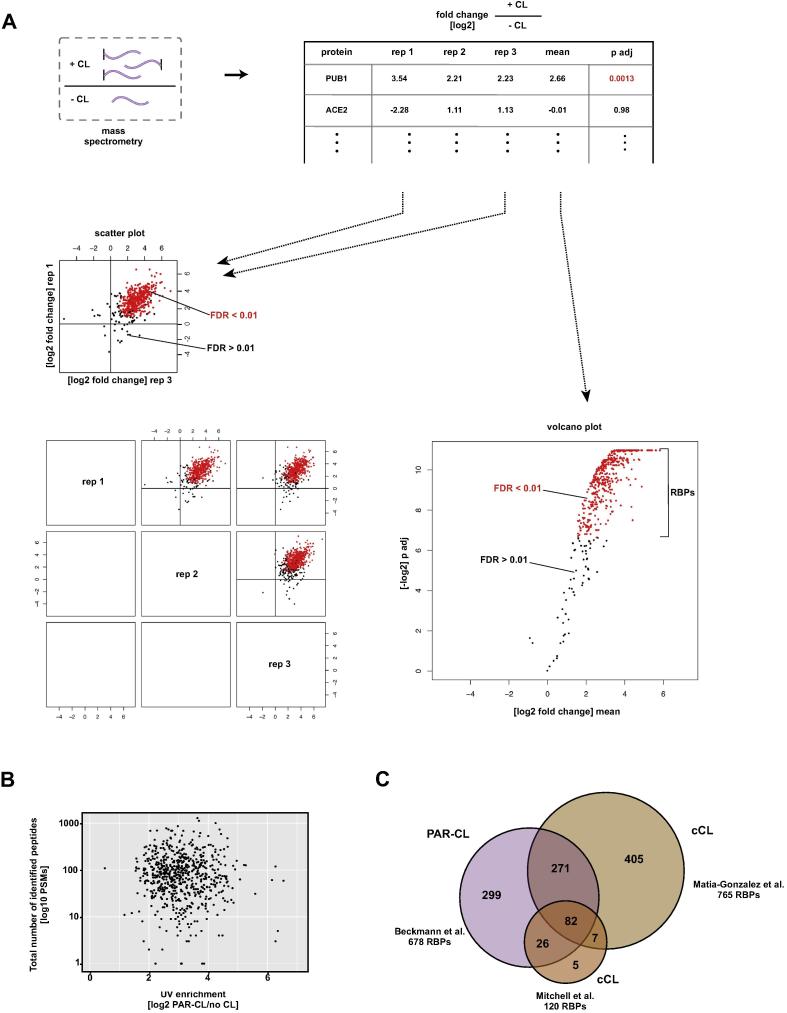
Comparison of yeast interactome capture to other studies. A) Schematic of a bioinformatic analysis of a high-throughput interactome capture experiment. Proteins enriched with a false discovery rate (FDR) of 0.01 or below represent the RNA interactome (red). B) Abundance of yeast RBPs [26] as total number of identified peptides (PSMs) is plotted against the enrichment of UV-crosslinked RBPs over the non-crosslink controls [5]. C) Venn diagram of identified RBPs from either PAR-CL [5] or cCL [44], [40]. (For interpretation of the references to colour in this figure legend, the reader is referred to the web version of this article.)

To rule out that highly abundant proteins are falsely selected as RBPs (potential false positives), we included staining for proteins such as tubulin and histones in our western blots, serving as negative controls since these proteins are not expected to bind to RNA (Fig. 2C,D). Additionally, we used the yeast RBP data obtained from [5] and compared the cellular levels of identified proteins (taken from [26]) with the enrichment in interactome capture recovery after crosslinking compared to the non-crosslinking control (Fig. 3B). We observe no correlation between overall protein abundance and enrichment in interactome capture, arguing against a favoured selection of abundant proteins in our protocol.

We and others have previously used 254 nm UV light to crosslink RNA and proteins in an RNA-labeling independent manner (cCL) [11], [35], [5]. Recently, two laboratories have used this approach to investigate RBPs in yeast in a similar manner [45], [40]. Comparison of interactome capture data originated from either cCL (120 RBPs [44]; 765 RBPs [40]) and PAR-CL (678 RBPs [5]) in yeast which yield 417 (cCL) and 299 (PAR-CL) RBPs exclusively (Fig. 3 C) indicates that the afforementioned features of RBPs might not play a pivotal role for capture of an RBP by interactome capture with a high enrichment score – or if it can be captured with one of the two irradiation techniques at all. The overlap between RBPs captured with both UV crosslinking approaches is only ∼35% which is rather low when compared to human interactomes in which 64% and 65% of the RBPs are detected using UV light at both wavelengths in HeLa cells [11] or HuH-7 cells [5], respectively. However, the yeast studies have been performed using different background correction (non-crosslink control vs. poly (A) RNA removal) and a different mass spectrometry approach which might contribute to the bigger diversity in commonly detected RBPs.

Taken together, none of the afforementioned features of RBPs such as abundance, localisation or domain composition of the purified RNA-binding proteins appear to dominate in the interactome capture process. Rather, UV crosslinking efficiency seems to be a main determinant for the outcome of the method which is also shown by the high dependance of individual proteins to be crosslinked and purified by one of the two irradiation methods (cCL or PAR-CL) exclusively. The technique of RNA-protein crosslinking utilising 4SU has been used from the 1980s on to study protein-binding to RNA and to map RNA:RNA interactions [21], [43], [18]. Unfortunately, despite the fact that the biophysical principles of the crosslinking are relatively well understood [20], the result of an *in vivo* UV crosslinking experiment remains a black box for the time being: to date, we cannot predict which individual RBPs will crosslink to individual transcripts or at which efficiency. High-throughput studies such as the yeast RNA interactome determined via either cCL [44], [40] or PAR-CL [5] thus hopefully help to guide researchers to use the appropriate crosslinking technique when investigating individual RBPs.
