# Supplementary material for: RNA interactome capture in yeast
Source: Methods. 2017 Apr 15;118-119:82–92. doi: 10.1016/j.ymeth.2016.12.008 (PMC5421583; doi:10.1016/j.ymeth.2016.12.008)
Supplement: Supplementary data [file mmc1.pdf]

Yeast mRNA interactome capture protocol

Benedikt Beckmann
Hentze group
EMBL Heidelberg, Germany

July 31, 2014

Contents

1 Considerations before you begin **1**

2 Cell culture and *in vivo* labeling **2**

3 *In vivo* crosslinking and cell lysis **2**

4 Bead preparation **3**

5 Binding and purification **3**

6 RNase treatment and concentration **4**

A Buffers **5**

B Troubleshooting **6**
B.1 Crosslinking efficiency 6
B.2 Quality control 6
B.3 Problems during purification 6
B.4 Timing 7

Abstract

This protocol describes a method to purify mRNA-binding proteins from yeast cells using *in vivo* crosslinking. After *in vivo* labeling of RNA using 4-thio-uracil, cells are subjected to UV irradiation at 365 nm wavelength, resulting in covalent crosslinks between RNA and proteins. Selective purification of mRNA is facilitated by using oligo-d(T) beads, binding to polyadenylated RNA. Several stringent wash steps and finally RNAse-digestion allow efficient purification of the crosslinked proteins.

1 Considerations before you begin

In vivo crosslinking in yeast is challenging compared to eukaryotic cell culture samples, but has been successfully applied [Granneman et al., 2009, 2010]. PAR-CL (the usage of 4-thio-modified nucleotide analogs in combination with UV light at 365 nm wavelength) has been performed in human cell culture [Hafner et al., 2010] but also in yeast [Creamer et al., 2011].

For *in vivo* labeling of RNA in yeast, we use 4-thio**uracil** which is dissolvable in DMSO. Unlike in tissue culture, samples containing 4-thiouracil do not have to be kept in darkness during growth or lysis as daylight is not leading to background crosslinking in yeast according to our observations. Please note that we use the following abbreviations:

- 4SU: 4-thiouridine (dissolvable in water, for cultured mammalian cell lines, e.g. HeLa)
- 4tU: 4-thiouracil (dissolvable in DMSO, for yeast)

Both compounds are suited for incorporation and will result in the same RNA-modification; however we had better success using 4tU in yeast.
Another detailed discussion of the protocol (for HeLa cells) is described by Castello et al. [Castello et al., 2013]

2 Cell culture and *in vivo* labeling

- Start a 5 ml YPAD preculture by inoculating with a colony from a plate (use freshly streaked yeast) and grow overnight
- Start a 3 times 1 liter $SC_{Ura120\mu M}$ culture from the preculture (starting OD_{600} should be 0.01–0.05) and grow to OD_{600} 0.4–0.5 at 30°C with 160–180 rpm
- Add 500 µM 4-thiouracil (4tU) (10 ml of a 50 mM stock)
- Let the cells grow and harvest after 3 hrs
- Pellet the cells by centrifugation (4,000 rpm, 15 min, 4°C)
- Resuspend the cells in 40 ml cold water

3 *In vivo* crosslinking and cell lysis

During these steps, keep the cells on ice whenever possible.

- Distribute the sample to 2 petri-dishes (on ice)
- Crosslink cells in a Stratalinker device emmiting UV light at 365 nm with a distance of $\sim$ 5 cm from the UV bulb
- Energy settings:
  - Set time: 3 min up to 30 min (3 times 10 min with 20 sec pauses in between) or
  - Set energy: Start from 0.7 J/cm^2 up to 7 J/cm^2
- Harvest the cells from the petri dish with a pipette
- Spin down the cells by centrifugation (4,000 rpm, 5 min, 4°C)
- Resuspend the pellet in 2 ml of lysis solution
- Distribute the mixture in 2–3 2 ml screw-capped tubes with $\sim$ 300 µl acid washed glass beads
- Use a FastPrep machine for cell disruption (6 m/s, 60 sec): 5 repetitions with 30 sec pause in between to allow cooling of the cells

- Clear the lysate by centrifugation (12,000 rpm, 2 min at 4°C) and transfer the supernatant to 50 ml tubes
- Remove a 10–50 µl aliquot as input control and freeze at -80°C
- Snap-freeze the rest in N_2 and store at -80°C

4 Bead preparation

- Take 1 ml oligo $d(T)_{25}$ magnetic beads (per litre of starting culture) and place in a magnetic separator to remove the supernatant
- Add 1 ml lysis buffer and let stand for 1 min at room temperature
- Remove the supernatant again and repeat this wash step 2 times

5 Binding and purification

- Increase the volume of the frozen eluate to 40 ml (for 3 liters of starting yeast culture or at least 25 ml if less starting material was used) using lysis buffer
- Use $\sim$ 1 ml cell lysate to resupend the beads, add the beads to the lysate and rotate for 1 h at 4°C
- Collect the beads by placing the tubes in a magnetic separator at 4°C , wait until all beads are efficiently concentrated at the tube wall (takes up to 30 min)
- Save the supernatant and store on ice
- Resuspend the beads in 40 ml lysis buffer and rotate for 5 min at 4°C
- Discard the supernatant and wash 2 times with each buffer (40 ml / 25 ml) at room temperature for 5 min:
  - Wash buffer 1
  - Wash buffer 2
  - Low salt buffer
- Elute the mRNP-complexes by adding 1 ml elution buffer and shake at 55°C for 10 min
- Remove a 50 µl aliquot as elution control and freeze at -80°C
- Re-activate the beads for a second round of binding and purification by washing three times with lysis buffer before adding to the supernatant from the first round and repeat the complete procedure

We usually perform 2–3 rounds of purification with the same beads. Elutions maybe pooled afterwards or analysed seperately. RNA and proteins are still covalently bound. For analysis of purified proteins, RNase treatment is necessary and for analysis of purified (m)RNA, proteinase K treatment is advisable.

6 RNase treatment and concentration

- Add 5 x RNase buffer to the elution fraction and adjust volume with water
- Add 0.5 µl RNase A (10 mg/ml) and 0.5 µl RNase T1 (100 U/µl) and incubate for 1 h at 37°C
- Load sample in 500 µl fractions onto an Amicon filter unit with a cut-off of 3 kD and centrifuge for 30 min at full speed in a table-top centrifuge; repeat until all sample is concentrated
- Load 400 µl 50 mM NaCl and centrifuge to remove residual buffer components
- Recover concentrated samples ($\sim$ 25 µl)

At this point, the purified proteins can be analysed by SDS-PAGE or proteomics techniques.

References

Alfredo Castello, Rastislav Horos, Claudia Strein, Bernd Fischer, Katrin Eichelbaum, Lars M. Steinmetz, Jeroen Krijgsveld, and Matthias W. Hentze. System-wide identification of rna-binding proteins by interactome capture. *Nature protocols*, 8(3):491–500, Mar 2013. ISSN 1750-2799. doi: 10.1038/nprot.2013.020. URL http://www.ncbi.nlm.nih.gov/pubmed/23411631.

T J Creamer, M M Darby, N Jamonnak, P Schaughency, H Hao, S J Wheelan, and J L Corden. Transcriptome-wide binding sites for components of the saccharomyces cerevisiae non-poly(a) termination pathway: Nrd1, nab3, and sen1. *PLoS Genet*, 7(10), Sep 2011. doi: 10.1371/journal.pgen.1002329. URL http://www.hubmed.org/display.cgi?uids=22028667.

L Dölken, Z Ruzsics, B Rädle, C C Friedel, R Zimmer, J Mages, R Hoffmann, P Dickinson, T Forster, P Ghazal, and U H Koszinowski. High-resolution gene expression profiling for simultaneous kinetic parameter analysis of rna synthesis and decay. *RNA*, 14(9):1959–1972, Sep 2008. doi: 10.1261/rna.1136108. URL http://www.hubmed.org/display.cgi?uids=18658122.

S Granneman, G Kudla, E Petfalski, and D Tollervey. Identification of protein binding sites on u3 snorna and pre-rrna by uv cross-linking and high-throughput analysis of cdnas. *Proc Natl Acad Sci U S A*, 106(24):9613–9618, Jun 2009. doi: 10.1073/pnas.0901997106. URL http://www.hubmed.org/display.cgi?uids=19482942.

S Granneman, E Petfalski, A Swiatkowska, and D Tollervey. Cracking pre-40s ribosomal subunit structure by systematic analyses of rna-protein cross-linking. *EMBO J*, 29(12):2026–2036, Jun 2010. doi: 10.1038/emboj.2010.86. URL http://www.hubmed.org/display.cgi?uids=20453830.

M Hafner, M Landthaler, L Burger, M Khorshid, J Hausser, P Berninger, A Rothballer, M Ascano, A C Jungkamp, M Munschauer, A Ulrich, G S Wardle, S Dewell, M Zavolan, and T Tuschl. Transcriptome-wide identification of rna-binding protein and microrna target sites by par-clip. *Cell*, 141(1):129–141, Apr 2010. doi: 10.1016/j.cell.2010.03.009. URL http://www.hubmed.org/display.cgi?uids=20371350.

Jessica Spitzer, Markus Hafner, Markus Landthaler, Manuel Ascano, Thalia Farazi, Greg Wardle, Jeff Nusbaum, Mohsen Khorshid, Lukas Burger, Mihaela Zavolan, and Thomas Tuschl. Par-clip (photoactivatable ribonucleoside-enhanced crosslinking and immunoprecipitation): a step-by-step protocol to the transcriptome-wide identification of binding sites of rna-binding proteins. *Methods in enzymology*, 539:113–161, 2014. ISSN 1557-7988. doi: 10.1016/B978-0-12-420120-0.00008-6. URL http://www.ncbi.nlm.nih.gov/pubmed/24581442.

A Buffers

- $SC_{Ura120\mu M}$ medium: 1x YNB with SD - uracil, 120 μM uracil f.c. added, 1% glucose
- oligo d(T) lysis buffer: 20 mM Tris pH 7.5, 500 mM LiCl, 0.5% LiDS, 1 mM EDTA, 5 mM DTT, 1 x protease inhibitor mix (EDTA-free, Roche), 1 mg/ml RNasin, 200 mM vanadyl-ribonucleoside-complex (VRC)
- oligo d(T) wash buffer 1: 20 mM Tris-HCl pH 7.5, 500 mM LiCl, 0.1% LiDS, 1 mM EDTA, 5 mM DTT
- oligo d(T) wash buffer 2: 20 mM Tris-HCl pH 7.5, 500 mM LiCl, 1 mM EDTA
- oligo d(T) low salt buffer: 20 mM Tris-HCl pH 7.5, 200 mM LiCl, 1 mM EDTA
- oligo d(T) elution buffer: 20 mM Tris-HCl pH 7.5, 1 mM EDTA
- 5 x RNase buffer: Tris-HCl pH 7.5, 75 mM NaCl
- oligo d(T) beads: NEB, Order no. S1419S

B Troubleshooting

B.1 Crosslinking efficiency

Determination of crosslinking efficiency is complex and varies from protein to protein (own observation).

RNA analysis Analyse total RNA using a NanoDrop or other UV-spectrophotometer at 260 nm wavelength. Measure RNA concentration of all elution fractions. Normally, the crosslinked fraction contains less RNA than non-crosslinked controls (factor in yeast $\sim$ 1.6)

RNA-labeling efficiency We used a dot-blot-based assay to evaluate 4tU-incorporation in RNA of yeast using the method described by Dölken and colleagues [Dölken et al., 2008]. Also, HPLC-based methods exist, e.g. as described by Spitzer et al. [Spitzer et al., 2014].

B.2 Quality control

During the purification, some indicators show if the crosslinking was successful. You can use these for in-process quality control.

QC1: Bead appearance Directly after the adding lysis buffer or wash buffer 1 (5), beads look dark/brownish.

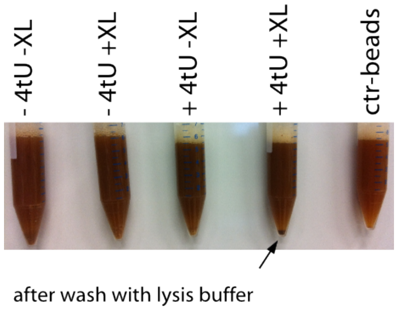

QC2: Bead appearance After washing with wash buffer 2 (5), a brownish "halo" appears around the magnetic beads when proteins were successfully crosslinked.

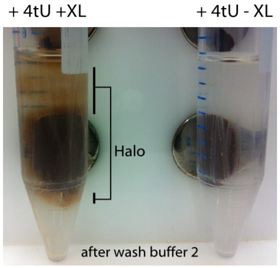

B.3 Problems during purification

White clouds/precipitates during binding and washes These white precipitates are most likely chromatin or DNA complexes. Increase the volume of buffer during the lysis and washes until they dissapear.

Brownish film on tube wall after low salt buffer washes or elution Beads sticking to the tube wall may impact efficiency of protein purification. One solution is to add NP40 to a final concentration of 0.05% to wash buffers 1 and 2. However, adding NP40 in too high concentrations or adding to the low salt buffer may render the elusions incompatible with any downstream mass spectrometry applications!

B.4 Timing

The protocol described here to purify mRBPs from yeast cells requires a lot of "hands-on" time and is usually done in three days:

- Day 1: cell growth, labeling, crosslinking and cell lysis
- Day 2: purification (2–3 rounds)
- Day 3: RNase digestion, concentration, SDS-PAGE, silverstaining or western blot

Two or three rounds of purification from 1–3 litre cultures usually takes a complete day which is mainly due to waiting time for complete magnetic bead separation. Moreover, during the many short-timed wash-magnet cycles during the washing steps, it becomes difficult to do other experiments in between.
